# Factors related to age at depression onset: the role of SLC6A4 methylation, sex, exposure to stressful life events and personality in a sample of inpatients suffering from major depression

**DOI:** 10.1186/s12888-021-03166-6

**Published:** 2021-03-25

**Authors:** Simon Sanwald, Katharina Widenhorn-Müller, Carlos Schönfeldt-Lecuona, Christian Montag, Markus Kiefer, GenEmo Research Group, Bernhard J. Connemann, Maximilian Gahr, Thomas Kammer

**Affiliations:** 1 Department of Psychiatry and Psychotherapy III, Ulm University, Ulm, Germany; 2 Institute of Psychology and Education, Department of Molecular Psychology, Ulm University, Ulm, Germany

**Keywords:** SLC6A4, DNA methylation, 5-HTTLPR, Stress, Major depression, Primary emotions, Age at onset

## Abstract

**Background:**

An early onset of depression is associated with higher chronicity and disability, more stressful life events (SLEs), higher negative emotionality as described by the primary emotion SADNESS and more severe depressive symptomatology compared to depression onset later in life. Additionally, methylation of the serotonin transporter gene (SLC6A4) is associated with SLEs and depressive symptoms.

**Methods:**

We investigated the relation of SLEs, SLC6A4 methylation in peripheral blood, the primary emotions SADNESS and SEEKING (measured by the Affective Neuroscience Personality Scales) as well as depressive symptom severity to age at depression onset in a sample of *N* = 146 inpatients suffering from major depression.

**Results:**

Depressed women showed higher SADNESS (*t* (91.05) = − 3.17, *p* = 0.028, *d* = − 0.57) and higher SLC6A4 methylation (*t* (88.79) = − 2.95, *p* = 0.02, *d* = − 0.55) compared to men. There were associations between SLEs, primary emotions and depression severity, which partly differed between women and men. The Akaike information criterion (AIC) indicated the selection of a model including sex, SLEs, SEEKING and SADNESS for the prediction of age at depression onset. SLC6A4 methylation was not related to depression severity, age at depression onset or SLEs in the entire group, but positively related to depression severity in women.

**Conclusions:**

Taken together, we provide further evidence that age at depression onset is associated with SLEs, personality and depression severity. However, we found no associations between age at onset and SLC6A4 methylation. The joint investigation of variables originating in biology, psychology and psychiatry could make an important contribution to understanding the development of depressive disorders by elucidating potential subtypes of depression.

**Supplementary Information:**

The online version contains supplementary material available at 10.1186/s12888-021-03166-6.

## Background

Despite the high prevalence of Major Depressive Disorder (MDD) [1], there has only been limited success in identifying reliable biomarkers [2]. Reasons for difficulties in the identification and replication of depression associated genetic risk loci are now gradually becoming apparent. One complicating factor is that depression is a polygenic disorder with single genes only explaining small amounts of variance [3, 4]. Second, depression has a high lifetime prevalence of about 15% [5]. Since depression is a common disorder showing one of the highest prevalences regarding psychiatric disorders, there is only a small mean difference in phenotypic liability between case/patient and control groups and thus reduced power to detect differences in allele frequencies between them [3]. Last, there is an ongoing debate about depression being one homogeneous clinical syndrome or a subsumption of distinct phenomenological entities under one diagnostic label [2]. Twin studies indicate that 45% of genetic liability to depression is not shared between sexes [6–8]. In addition, it has been shown that subgrouping depression according to recurrence or early age at depression onset yielded higher heritability estimations in case of recurrent depressive episodes and early onset depression [9, 10].

Early-onset depression may also differ from late-onset depression with respect to the course and symptoms of the disorder: Patients who had their first depressive episode early in life showed a shorter time to relapse and more residual symptoms after recovery [11]. Moreover, they reported higher chronicity and disability [12], had experienced more stressful life events (SLEs) and differed in depressive symptomatology from patients with adult-onset depression [13]. High scores on the personality dimension neuroticism is considered a vulnerability factor associated with early age at depression onset [13–15]. The association of depression onset with SLEs and neuroticism is in line with a theory of depression development taking into account mammalian-brain emotional systems, bridging the gap between a dysregulation of the bodily stress system and affective changes of depression [16]. In short, this theory suggests a stressor to cause initially elevated efforts to terminate the stress response. When termination fails, a behavioral shutdown protects the individual against the fatal consequences of wasting too much resources but leaving it with lassitude and despair [17]. This theory builds upon the framework of affective neuroscience, a term coined by Panksepp [18]. Affective neuroscience theory (see also [19]) promotes the view that human personality is anchored in primary emotional systems comprising the fundamental emotional tendencies common among all mammals [20–22]. According to the above theory of depression development, depression is mainly associated with two primary emotions: SEEKING and SADNESS [16]. The SEEKING system is utilized by other primary emotions and is defined as the effort made to mitigate a negative emotional state or the search for vital resources. SADNESS on the other hand is triggered by separation distress such as the loss of a child or being apart from a loved one (in humans the separation or loss may also be a symbolic one) and eventually precipitates depression [20]. Both, low SEEKING and high SADNESS are associated with depression severity [23] but whether they are associated with age at depression onset has, to the best of our knowledge, not yet been investigated.

At the same time the serotonin (5-HT) system has a long history in the research on the pathophysiology of affective disorders [24]. Early theories of depression development postulate depression to originate from a dysfunction of monoaminergic neurotransmission. More recent theories suggest a modulating and not a causal role of 5-HT in the etiology of depression [24]. Considering its important role for early theories of depression development, it is not surprising that the 5-HT system has often been investigated in search for genetic markers of depression risk. The serotonin transporter (5-HTT) is one of the most investigated parameters of the 5-HT system in depression research [25, 26]. 5-HTT is one of several structurally similar transporter proteins having a high affinity for monoamines. These presynaptic membrane proteins transport their substrate from the extracellular space to the cytoplasm terminating serotonergic neurotransmission and recycling presynaptic supplies of serotonin [27]. The interest in the 5-HTT originates from antidepressants such as selective serotonin reuptake inhibitors (SSRIs) directly binding to 5-HTT inhibiting 5-HT reuptake [28]. As a consequence of SSRI treatment, 5-HTT is highly regulated and undergoes adaptive changes [29]. The SLC6A4 gene encoding the 5-HTT, is located on chromosome 17 q.11.1-q12 [30], has a length of 31 kilo bases and contains 14 exons [31]. The serotonin transporter linked polymorphic region (5-HTTLPR) – a repeat length polymorphism – is one of the most investigated polymorphisms in depression research [32]. The short (S) allele is associated with a lower transcription rate of the 5-HTT compared with the long (L) allele [33]. Former reports of carriers of the S-allele being more likely to develop MDD as a function of SLEs (and sometimes sex) could not be confirmed in a recent meta-analysis [32].

However, in the last years another field of research emerged impressively demonstrating that the transcriptional apparatus and thus the phenotype is not hard-wired by the genome. In fact, the genome is the individual starting point for adaptation processes becoming necessary during cellular differentiation and due to individually different environmental challenges. The name epigenetics became established as term for processes bridging the gap between genotype and phenotype [34]. The most investigated epigenetic mechanism is DNA-methylation [35]. DNA-methylation has mostly been examined in cytosine-phosphate-guanine (CpG) dinucleotides. In CpG dinucleotides a methyl group is added to the 5′-position of the cytosine residue [35]. CpG-methylation affects histone DNA interactions thereby modulating a gene’s accessibility for transcription factors and thus transcriptional activity [36, 37]. This is not surprising in light of the fact that CpG rich regions are frequently located in the promoter regions of genes [38].

Increased methylation of the whole CpG island or specific CpG sites in the promoter region of the SLC6A4 gene has already been associated with decreased transcription rate [39], lower mRNA concentrations [40], SLEs and recent depressive symptoms [41, 42], family history of depression [43] as well as post-stroke depression [44]. There are, however, ambiguous results regarding the association between SLC6A4 promoter methylation and depression severity [42, 43, 45–47]. In addition, there are reports of an interaction of 5-HTTLPR genotype and DNA-methylation in the investigation of the effects of stress on stress-related phenotypes [40, 48]. Thus, variations in SLC6A4 expression need to be integrated with the contribution arising from genetic as well as epigenetic mechanisms [49]. However, DNA methylation may also be sex specific. Especially in depression with its sexually dimorphic risk for depression development [50]. Accordingly, CpG sites in the SLC6A4 gene have been reported to be differentially methylated as a function of sex with females exhibiting higher SLC6A4 methylation than men [51]. Therefore, sex by genotype interactions should be explored when investigating SLC6A4 methylation in depression.

Taken together, depression can be understood as reduced SEEKING/higher SADNESS as a consequence of chronically prolonged separation distress [16]. The 5-HT system plays an important role in affective disorders [52] with the 5-HTT being the target for a large group of antidepressants [28]. Furthermore, SLEs are associated with SLC6A4 methylation and early-onset depression [13, 41]. Early-onset depression has been shown to have a higher heritability compared to the investigation of depression independent of age at onset [10]. There are findings of interactions between sex and genetic as well as epigenetic layers of SLC6A4 regulation [40, 48, 51]. Thus, we wanted to examine the relation of SLEs, primary emotions, DNA-methylation of SLC6A4 and their interactions to age at depression onset in a sample of inpatients suffering from MDD. We assumed depression onset to be positively associated with SLEs, SADNESS and depression severity and negatively associated with SEEKING. We also assumed an association between age at onset and SLC6A4 methylation. Considering the heterogeneity of findings in previous studies, we did not infer a directional hypothesis for associations between age at onset and SLC6A4 methylation. In addition, we wanted to explore possible sex differences between SLEs, primary emotions, age at depression onset, SLC6A4 methylation and depression severity. Last, we wanted to examine which factors (sex, SLEs, primary emotions, 5-HTTLPR genotype and SLC6A4 methylation) or interactions of factors predict age at depression onset.

## Methods

### Participants

Data of *N* = 146 inpatients (*n* = 95 females, age: *M* = 38.74, *SD* = 14.25) diagnosed for major depression at the time of admission to the hospital was taken from the database of the Ulm Gene Brain Behavior Project (UGBBP). Data from this sample was used for earlier studies focusing on other parameters [23, 53, 54]. All inpatients were recruited at the Department of Psychiatry and Psychotherapy III at Ulm University, Ulm, Germany. They were diagnosed for Major Depression by a psychiatrist at admission to the hospital using the Structured Clinical Interview for DSM-IV (SCID-I) [55]. Participants were administered the self-assessment questionnaires described below. Depression severity was rated by a trained interviewer using the Montgomery Asberg Depression Rating Scale (MADRS) [56]. Sociodemographic data was collected with a standardized semi-structured interview based on an in-house questionnaire. Further, we assessed age, Body Mass Index (BMI in kg/m^2^), consumed alcohol (grams/day) and nicotine (cigarettes/day). Patients were asked when they had had their first episode of at least 2 weeks suffering from depressive mood, loss of interest and other depressive symptoms. We calculated dose equivalents for current antidepressants (weighted mean dose/fluoxetin40 mg) [57] and neuroleptics (weighted mean dose/chlorpromazine100 mg) [58–60]. Two patients fulfilled the DSM-IV criteria [55] for alcohol abuse but not alcohol dependence. We decided to include them in our analyses since we controlled for alcohol consumption. One patient had the diagnosis of a sexual dysfunction not otherwise specified. 56.8% of inpatients (*n* = 83) reported to know about the presence of psychiatric disorders in their family (depression: *n* = 56 with *n* = 38 in parents or siblings and *n* = 18 in more distant relatives or not otherwise specified; schizophrenia: *n* = 5 with *n* = 2 in parents or siblings; anxiety: *n* = 4 with *n* = 2 in parents or siblings; personality disorders: *n* = 3 with *n* = 1 in siblings; bipolar disorder: *n* = 2 in parents or siblings; eating disorders: *n* = 3 in more distant relatives; substance abuse: *n* = 2 with *n* = 1 in both parents and siblings; obsessive compulsive disorder: *n* = 1 in parents; attention deficit syndrome: *n* = 1 in siblings; not otherwise specified: *n* = 19). *Median* age at depression onset was 21 years. Please note that there is an overlap between the present manuscript and older publications [23], where the ANPS has been investigated in the context of BDI scores in a case-control design (but without SLEs and epigenetic variables).

### Questionnaires

#### CLEQ

The Critical Life Events Questionnaire (CLEQ) assesses 30 traumatic life events such as sexual abuse, experience of violence or death of a close person. The participants answered a question of whether they had ever experienced the concerning event [61]. We calculated a score adding up the experienced events. If there were 9 or more unanswered events, participants were excluded from further analysis with the CLEQ.

#### Affective neuroscience personality scales (ANPS)

The ANPS German version [62] comprises 110 items assessing individual tendencies in six primary emotional systems: SEEKING, CARE, PLAY (positive emotionality) and FEAR, ANGER, SADNESS (negative emotionality). The primary emotion of LUST may potentially have negative carry over effects on the remaining items, if items on one’s own sexual behavior would be filled in. All items are answered on a four point Likert scale ranging from strongly disagree [1] to strongly agree [4]. Internal consistency of the SEEKING scale was acceptable (*α* = .77, *n* = 110, *n* = 36 were excluded listwise), internal consistency of the SADNESS scale was also acceptable (*α* = .73, *n* = 116, *n* = 30 were excluded listwise).

#### BDI-II

Severity levels of depressive symptoms were explored by using the Beck Depression Inventory (German version, BDI-II) [63]. The BDI-II is an internationally recognized clinical and research psychopathological-psychometric instrument recording the severity of a depressive syndrome. The BDI-II is a self-assessment scale and comprises 21 items. For each item ratings between 0 (not at all) and 3 (very intensive) are given depending on the symptom severity. A maximum of 63 points in total can be reached. Internal consistency was good with *α* = .84.

### Genotyping of 5-HTTLPR

DNA extraction from whole blood samples was performed on MagNA Pure 96 using a commercial extraction kit (Roche, Mannheim, Germany). Genotyping of the 5-HTTLPR including rs25531 was carried out as described in Lachmann and colleagues [64]. The combination of information from the 5-HTTLPR and rs25531 results in the distinction between the variants L_A_ and L_G_. L_G_ is functionally similar to the S allele [65]. Frequencies of 5-HTTLPR/rs25531 genotype fulfilled Hardy-Weinberg-Equilibrium expectations with respect to 5-HTTLPR (49 L/L, 72 L/S, 23 S/S; *χ*^2^(1) = 0.16, *p* = 0.69). To maximize statistical power, groups were dichotomized into L_A_L_A_ homozygotes and S/L_G_ carriers (42 L_A_L_A_, 102 S/L_G_).

### Quantification of SLC6A4 promoter methylation

**Fig. 1:**
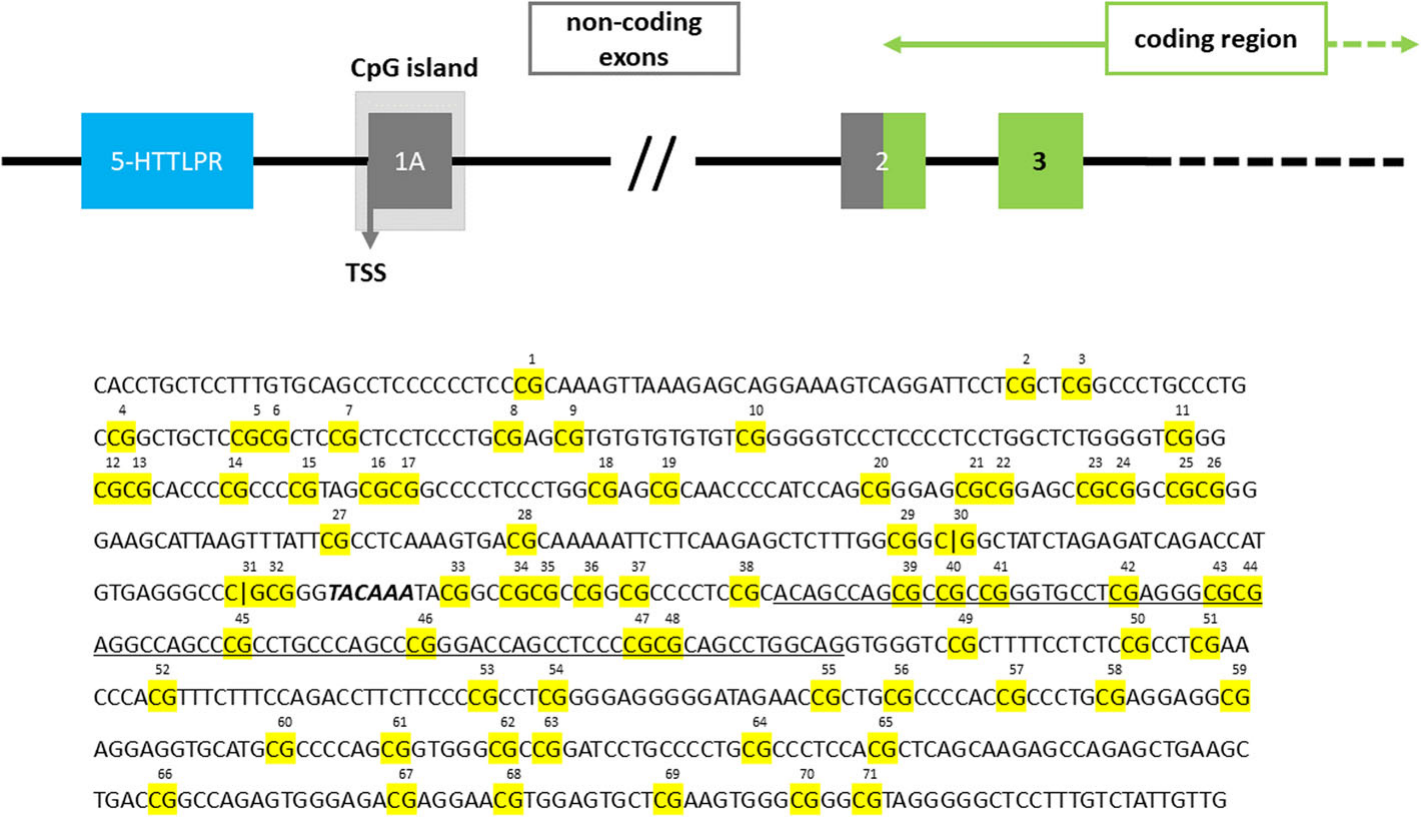
Schematic representation of the SLC6A4 gene. The position of the examined CpG island is marked by a light grey box. CpG sites are marked in yellow color and numbered. Bold and italic letters mark the putative TATA-box. Exon 1A is underlined

Methylation status of the SLC6A4 gene (Fig. 1) was quantified by Varionostic GmbH (Ulm, Germany) using the Sequenom Epityper MassArray System (San Diego, CA, USA). All steps of the EpiTYPER assay were performed under routine conditions as described by Suchiman and colleagues [66]. Genomic DNA from peripheral blood was bisulfite treated. Amplicons for the CpG-rich region in the SLC6A4 promoter (chr17:30235345–30,236,068; amplicon 1: chr17:30235345–30,235,765; amplicon 2: chr17:30235734–30,236,068; hg38) were designed using Agena’s EpiDESIGNER software (San Diego, CA, USA). These amplicons were PCR amplified using the following primers: amplicon 1: forward (aggaagagagG GTTATTTAGAGATT AGATTATGTGAGGGT) and reverse (cagtaatacgactcactatagggagaaggctCA ACAATAAACAAAAAAACCCCCTA); amplicon 2: forward (aggaagagagG GGTTTTTATATGGTTTGATTTTTAG) and reverse (cagtaatacgactcactatagggagaaggctCACCTACTCCTTTATACAACCTCCC).

In a next step, in-vitro RNA transcription with subsequent base-specific cleavage using RNase A was performed. This procedure resulted in fragmented RNA molecules of identical length. RNA molecules differed in their nucleotide composition due to bisulfite treatment. After sample preparation, a MALDI-TOF platform (Agena; MassArray 4) was used to process the probes. Resulting data from the mass spectrometer was preprocessed using the EpiTYPER Analyser. Methylation status was quantified by analyzing the mass spectra.

**Fig. 2:**
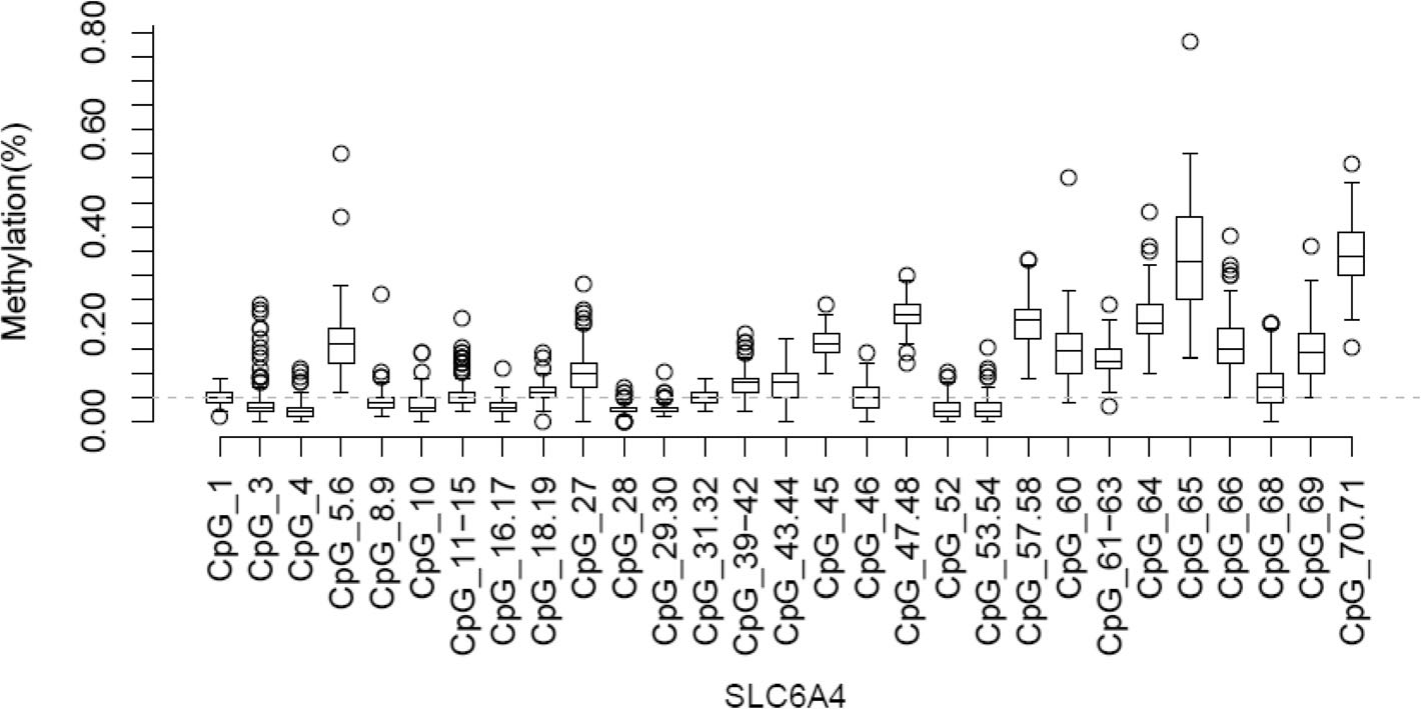
Boxplots for the methylation status of the examined CpG Sites in the SLC6A4 CpG island

We assessed methylation status regarding single CpG units (some CpG sites lay on the same RNA fragment and methylation status reflects the mean of all sites on this fragment). Boxplots for CpG units with analyzable methylation status are depicted in Fig. 2. Since we wanted to have a joint measure of SLC6A4 DNA methylation, we first analyzed the associations between all CpG sites and units (see the supplementary material, Table S1). Reliability across CpG sites was good (*α* = .82). However, there were very low correlation coefficients between some of the CpG units. Therefore, we performed a Principal Component Analysis (PCA) to extract the most important independent factors for SLC6A4 methylation. The Kaiser–Meyer–Olkin measure of sampling adequacy was .698, representing a relatively good factor analysis, and Bartlett’s test of sphericity was significant (*p* < .001). Only factors explaining more than 10% of variance in the methylation data were considered [67], resulting in a two factor solution. The two factors accounted for 31.44% of the total variance in SLC6A4 methylation. Among the factor solutions, the varimax-rotated two-factor solution yielded the most interpretable solution (factor 1: centric to terminal CpG units of the investigated region; factor 2: anterior and centric CpG units), and most CpG units loaded highly on only one of the two factors (for further information and the varimax-rotated two factor solution see supplementary material Tables S2). These two factors of SLC6A4 methylation were entered in the statistical analyses.

### Statistical analysis

Statistical analyses were conducted using *R* [68] with the packages *psych* [69], and *ggplot2* [70] as well as IBM *SPSS* Statistics for Windows, version 25. To control for confounding variables associated with SLC6A4 methylation or age at depression onset, we tested associations between potential covariates (age, BMI, substance use, dose equivalents of antidepressants and neuroleptics) and SLC6A4 methylation as well as age at depression onset using Spearman’s correlation coefficients. Next, we investigated sex differences and differences between inpatients without and with a family history of psychiatric disorders performing Welch’s *t*-tests (note that U-tests provided similar results). Thereafter, we calculated partial Spearman’s correlation coefficients between the variables of interest controlling for potentially confounding variables. In detail, we examined associations between depression severity (BDI-II and MADRS), CLEQ score, SEEKING, SADNESS, age at depression onset as well as both factors of SLC6A4 methylation. In addition, we wanted to explore, which of the examined variables is a predictor of depression onset and whether interactions between sex, 5-HTTLPR genotype and SLC6A4 methylation affect early depression onset. Thus, we performed a stepwise hierarchical linear regression analysis with sex, CLEQ, SEEKING, SADNESS, 5-HTTLPR genotype (L/L vs. S+), SLC6A4 factor 1, SLC6A4 factor 2 as well as interactions between DNA methylation and sex as wells as 5-HTTLPR genotype as predictors in the full model. Age at depression onset was the dependent variable. We used an automatic stepwise model selection by the Akaike information criterion (*AIC*) allowing for iteratively adding and removing predictors. Benjamini-Hochberg correction was used controlling false discovery rate (FDR) [71]. Statistical significance was determined at *p* < .05; all tests were two-tailed.

## Results

### Control analyses

Age and BMI were significantly associated with SLC6A4 methylation and/or age at depression onset (Table 1). Therefore, age and BMI were added as covariates in all analyses reported below. 5-HTTLPR genotype groups did not differ in SLC6A4 methylation neither for methylation factor 1 (L_A_L_A_: *n* = 35, *M* = 0.06, *SD* = 1.04; S/L_G_: *n* = 91, *M* = − 0.08, *SD* = 0.93; *t* (56.06) = 0.68, *p* = .50) nor for methylation factor 2 (L_A_L_A_: *n* = 35, *M* = − 0.22, *SD* = 0.09; S/L_G_: *n* = 91, *M* = 0.09, *SD* = 1.08; *t* (73.96) = − 1.62, *p* = .11) nor for age at depression onset (L_A_L_A_: *M* = 26.02, *SD* = 12.72; S/L_G_: *M* = 25.78, *SD* = 13.44; *t* (80.49) = 0.11, *p* = .92). There were no significant genotype differences in any of the investigated variables even before controlling FDR (see supplementary material Table S4).

**Table 1:** Spearman’s correlation coefficients between SLC6A4 methylation as well as age at onset and potentially confounding variables

		SLC6A4 factor 1	SLC6A4 factor 2	age at onset
Age	*r*	0.41	0.21	0.60
	*p*	0.000	0.019	0.000
BMI	*r*	0.13	0.15	0.21
	*p*	0.159	0.105	0.012
alcohol (grams/day)	*r*	0.01	0.01	0.07
	*p*	0.904	0.931	0.415
cigarettes/day	*r*	−0.05	−0.17	−0.04
	*p*	0.604	0.052	0.660
DE antidepressants	*r*	−0.14	0.10	0.13
	*p*	0.165	0.324	0.149
DE neuroleptics	*r*	0.05	−0.14	0.05
	*p*	0.623	0.110	0.585

*DE* dose equivalents. Spearman’s correlation coefficients. *P*-values not FDR corrected

### Sex differences and differences between inpatients without and with a family history of psychiatric disorders

The investigation of sex differences revealed women to score higher than men with respect to the primary emotion SADNESS. Furthermore, women showed higher methylation of centric to terminal CpG sites as indexed by factor 1 of SLC6A4 methylation. There were no sex differences in any of the other variables. Exact descriptive and inferential statistics can be found in (Table 2). There were no significant differences between inpatients without and inpatients with a family history of psychiatric disorders after FDR correction (Table 3).

**Table 2:** Sex differences in the covariates and variables of interest

	*n (m/f)*	*M*_*men*_	*SD*_*men*_	*M*_*women*_	*SD*_*women*_	*t*	*df*	*p*_*BH*_	*d*
Age	51/95	39.29	13.98	38.44	14.46	0.35	105.44	0.785	0.06
BMI	51/95	26.48	4.68	25.66	7.02	0.85	137.30	0.557	0.14
alcohol (grams/day)	49/89	7.72	20.76	2.06	6.69	1.86	53.55	0.193	0.37
cigarettes/day	50/94	6.96	10.53	4.62	7.18	1.41	73.81	0.306	0.26
DE antidepressants	46/78	41.66	31.08	33.81	26.93	1.43	84.04	0.306	0.27
DE neuroleptics	50/93	22.50	44.10	27.15	49.54	−0.58	110.86	0.634	− 0.10
BDI-II	50/89	29.61	12.16	33.74	10.58	−2.01	90.44	0.162	−0.36
MADRS	51/95	24.00	9.64	25.66	9.95	−0.98	105.18	0.534	−0.17
CLEQ	48/90	7.71	5.25	7.82	4.37	−0.13	82.16	0.898	−0.02
SEEKING	48/89	2.38	0.37	2.43	0.40	−0.74	104.04	0.569	−0.13
SADNESS	49/89	2.80	0.39	3.02	0.35	−3.17	91.05	0.028	−0.57
age at onset	51/95	29.43	14.31	23.80	12.05	2.39	88.47	0.089	0.43
SLC6A4 factor 1	46/81	−0.38	0.97	0.13	0.91	−2.95	88.79	0.028	−0.55
SLC6A4 factor 2	46/81	0.20	1.16	−0.12	0.95	1.58	79.43	0.280	0.30

Welch’s *t*-test

**Table 3:** Differences between inpatients without (no) and with (yes) family history of psychiatric disorders

	*n (no/yes)*	*M*_*no*_	*SD*_*no*_	*M*_*yes*_	*SD*_*yes*_	*t*	*df*	*p*_*BH*_	*d*
Age	44/83	41.68	12.93	37.51	14.31	1.67	95.74	0.453	0.31
BMI	44/83	26.96	6.56	25.60	6.18	1.13	83.27	0.453	0.21
alcohol (grams/day)	42/78	7.25	22.07	2.85	7.78	1.25	46.56	0.453	0.27
cigarettes/day	44/81	4.80	6.70	6.03	9.61	− 0.84	115.37	0.566	−0.15
DE antidepressants	35/73	35.05	21.48	37.43	31.67	−0.46	93.71	0.697	−0.09
DE neuroleptics	43/81	24.42	51.90	29.32	47.73	−0.52	79.75	0.697	−0.10
BDI-II	43/79	30.21	10.78	33.09	12.08	−1.35	95.12	0.453	−0.25
MADRS	44/83	23.27	9.49	25.59	9.77	−1.30	90.05	0.453	−0.24
CLEQ	43/80	7.28	4.33	8.21	5.19	−1.06	100.09	0.453	−0.20
SEEKING	42/80	2.41	0.31	2.43	0.42	−0.33	107.29	0.745	−0.06
SADNESS	43/80	2.88	0.40	2.96	0.38	−1.11	82.57	0.453	−0.21
age at onset	44/83	29.43	13.12	23.75	12.74	2.35	85.53	0.294	0.44
SLC6A4 factor 1	37/72	0.04	1.10	−0.06	0.83	0.48	58.08	0.697	0.10
SLC6A4 factor 2	37/72	0.22	0.99	−0.17	1.06	1.85	77.27	0.453	0.37

Welch’s *t*-test

### Correlation analyses

Partial Spearman’s correlation analyses of the whole sample (Table 4) showed that after FDR correction there was a significantly positive association between CLEQ and depression severity (with both BDI-II and MADRS) as well as SADNESS. In addition, the CLEQ was significantly negatively associated with age at depression onset. SEEKING was significantly negatively associated with depression severity (with both BDI-II and MADRS) and SADNESS. SADNESS on the other hand was significantly positively associated with depression severity (with both BDI-II and MADRS) and significantly negatively associated with age at depression onset. Besides the significantly negative associations between age at onset and CLEQ as well as SADNESS, age at onset was also significantly negatively associated with depression severity (BDI-II only).

**Table 4:** Partial Spearman’s correlation coefficients (below the diagonal) and *p*-values (above the diagonal for the whole sample and both sexes separately

whole sample (***N*** = 146)	BDI-II	MADRS	CLEQ	SEEKING	SADNESS	age at onset	SLC6A4 factor 1	SLC6A4 factor 2
BDI-II		< 0.001	< 0.001	< 0.001	< 0.001	0.025	0.700	0.700
MADRS	0.62***		0.035	< 0.001	< 0.001	0.160	0.700	0.123
CLEQ	0.33***	0.21*		0.700	0.011	0.001	0.869	0.598
SEEKING	−0.32***	− 0.32***	0.05		0.008	0.102	0.998	0.416
SADNESS	0.49***	0.42***	0.25*	−0.26**		< 0.001	0.998	0.755
age at onset	−0.22*	−0.14	− 0.31**	0.17	−0.35***		0.220	0.700
SLC6A4 factor 1	0.06	0.05	0.02	−0.00	− 0.00	− 0.14		0.372
SLC6A4 factor 2	−0.05	0.17	−0.08	0.10	0.04	0.05	− 0.11	
**women** **(*n*** **= 95)**								
BDI-II		< 0.001	0.065	0.006	< 0.001	0.437	0.479	0.998
MADRS	0.66***		0.562	0.012	< 0.001	0.851	0.851	0.026
CLEQ	0.25	0.10		0.826	0.769	0.042	0.361	0.479
SEEKING	−0.36**	− 0.33*	0.05		0.026	0.361	0.945	0.479
SADNESS	0.47***	0.47***	0.07	−0.30**		0.049	0.479	0.785
age at onset	−0.14	−0.04	− 0.27*	0.16	−0.26*		0.769	0.907
SLC6A4 factor 1	−0.13	−0.04	− 0.17	0.01	−0.13	0.07		0.945
SLC6A4 factor 2	−0.00	0.31*	−0.12	0.13	0.06	0.03	− 0.02	
**men** **(*n*** **= 51)**								
BDI-II		< 0.001	0.014	0.180	0.006	0.181	0.829	0.956
MADRS	0.61***		0.011	0.124	0.047	0.047	0.956	0.956
CLEQ	0.45*	0.46*		0.975	0.001	0.026	0.401	0.956
SEEKING	−0.27	− 0.30	0.00		0.578	0.101	0.962	0.956
SADNESS	0.50**	0.36*	0.56**	−0.15		0.014	0.956	0.703
age at onset	−0.26	−0.36*	− 0.40*	0.32	−0.43*		0.233	0.956
SLC6A4 factor 1	0.09	0.05	0.20	−0.02	−0.05	− 0.25		0.616
SLC6A4 factor 2	−0.02	−0.02	0.06	0.04	0.12	− 0.03	−0.14	

Covariates: age and BMI. FDR corrected *p*-values (for each sample separately). *** *p* < .001, ** *p* < .01, * *p* < .05

Analysis of both sexes separately revealed similar result patterns. However, the significantly positive associations between CLEQ and depression severity as well as SADNESS was present only in men. In addition, men as compared to women showed medium size negative associations between age at depression onset and depression severity. This association was significant for the MADRS. In women, we found a significantly positive association of medium size between methylation factor 2 of SLC6A4 and depression severity (MADRS only). This association was not present in men.

For associations between single CpG sites/units and depression severity, CLEQ, SEEKING, SADNESS and age at onset, see the Supplementary Material (Table S3).

### Hierarchical linear regression analysis

In order to determine which predictors explain a significant amount of variance considering age at depression onset, we performed a stepwise regression analysis (both directions). We included age and BMI as covariates and sex, CLEQ, SEEKING, SADNESS, 5-HTTLPR genotype, SLC6A4 factor 1, SLC6A4 factor 2 as well as interactions between DNA methylation, sex and 5-HTTLPR genotype as predictors in the full model. All metric variables were standardized. The Akaike information criterion (AIC) indicated the selection of a model including sex, SLEs, SEEKING and SADNESS for the prediction of age at depression onset. This model is shown in Table 5 and explained a significant amount of variance in age at depression onset (*R*^*2*^ = 0.60, *F* (6,110) = 29.44, *p* < .000001). Higher age at the time of measurement was associated with lower age at depression onset. Female sex and experience of SLEs were associated with lower age at depression onset. Higher SEEKING was associated with higher age at depression onset.

**Table 5:** Final model of the stepwise regression analysis with age at onset as dependent variable

Predictor	*b*	*SE*	*t*	*p*
(Intercept)	0.49	0.22	2.21	0.029
Age	0.69	0.06	10.75	0.000
BMI	−0.06	0.06	−0.93	0.356
Sex	−0.30	0.13	−2.30	0.024
CLEQ	−0.16	0.06	−2.61	0.010
SEEKING	0.13	0.06	2.09	0.039
SADNESS	−0.11	0.07	−1.57	0.119

The covariates age and BMI were included by default

## Discussion

In the present study, we examined the role of SLEs, primary emotions, DNA-methylation of SLC6A4 and depression severity for depression onset. We assumed early-onset depression to be associated with more SLEs, less SEEKING, higher SADNESS and higher depression severity. We also assumed an association between age at onset and SLC6A4 methylation. In addition, we wanted to examine which variables might serve as best predictors explaining a significant amount of variance in age at depression onset. Basing on previous findings, we also investigated possible SLC6A4 methylation × sex × 5-HTTLPR interactions in the investigation of depression onset.

As expected, we found depression onset to be negatively associated with SLEs after controlling for age and BMI. This is in line with previous results of more SLEs in patients with early as compared to patients with adult-onset depression [13] and with the association of stress with first depression onset [72, 73]. The association between stress and depression is a well-established finding that has been documented for various stressors [74, 75], recent and early SLEs [76, 77] and in a variety of samples with different age groups [53, 78, 79]. In the adolescent period, the individual could be especially vulnerable to stressors since this period is considered important for the organization of behavioral and endocrine responses to stress. In addition, the maturation of brain systems involved in the control of the HPA-axis takes place during this transition period [80]. It is possible that inpatients with an early depression onset had an adverse environment in early developmental stages [81]. However, as data about early life adversities was not available in the present sample, this hypothesis needs to be tested in future studies.

SLEs in these early developmental stages could be a predictor of current depressive symptom severity because of their long lasting effects on the bodily stress system. Daskalakis and colleagues [82] suggest a three-hit concept of vulnerability and resilience in the face of early life adversity: They postulate an interaction of genetic factors (hit-1) with early environmental factors (hit-2) to be reflected in epigenetic modifications and altered endocrine regulations. This interaction programs gene expression patterns, which are relevant for an evolving phenotype during brain development. The emerging phenotype with altered stress axis regulation and sensitivity is exposed to the later-life environment (hit-3). Depending on the type of later-life challenge, the individual is either vulnerable or resilient to the development of psychiatric symptoms [82]. The three-hit concept provides a framework for the interpretation of early as well as later-life SLEs’ association with depressive symptoms [83] and takes into consideration why some individuals develop depression after the experience of early adversity and some do not. This in turn is an explanation for the finding of a medium size positive association between SLEs and depressive symptoms, which is in line with previous studies [13].

High SADNESS scores were associated with younger age at depression onset. This is in line with previous studies reporting high scores on the personality dimension neuroticism to be a vulnerability factor for early depression onset [13, 14]. However, after taking into account other predictors, SADNESS did not explain a significant amount of variance in age at depression onset. An explanation could be that there are associations between SLEs and SADNESS, between SEEKING and SADNESS and between sex and SADNESS. Thus, SADNESS shares variance with three of the other predictors included in the final model. However, SADNESS was still included in the model with the lowest AIC.

While there was a small to medium size positive correlation coefficient between SEEKING and age at depression onset, this association was not significant. In the regression model predicting age at onset, however, SEEKING explained a significant amount of variance in the dependent variable beyond the variance explained by other predictors. Thus, looking at the model best suited for the prediciton of age at depression onset, our findings are in line with the theory of depression development by Watt and Panksepp [16]. Therefore, we want to give a possible explanation for our findings with reference to their theory: If an individual has high SADNESS scores arising from social loss or defeat [17], low reward-SEEKING protects against new social losses or defeats. However, social isolation also prevents positive social experiences, which in turn can be considered a social loss if it is a permanent condition. This vicious circle might culminate in a major depressive episode in line with Lewinsohn’s [84] social reinforcement theory. Therefore, SLEs, SADNESS and SEEKING might serve for early identification of individuals at risk of developing depression. Since the ANPS provides the opportunity for detecting individuals scoring high on two main symptoms of depression, depressive mood and loss of interest/energy, it could be better suited for the detection of individuals at risk for depression development than examining Neuroticism only. Of note, Neuroticism represents a super-factor, being not only associated with SADNESS, but also FEAR and ANGER [20, 22]. In terms of Affective Neuroscience theory, SADNESS should be the core dimension to understand depression, although strong overlaps with FEAR have been observed aswell [23]. At this point we emphasize that it is not clear whether associations between age at onset or depression severity and SEEKING as well as SADNESS are an expression of primary emotions predisposing for depression or for depression having an impact on primary emotions. This needs to be evaluated using a longitudinal design.

Our findings are not only in line with interventions used for the treatment of depression, they could additionally provide an evolution based and easy to understand explanation of why these interventions are effective. For instance, behavioral activation is an effective initial intervention for the treatment of depression [85]. This may be explained by an activation of the downregulated SEEKING system thereby enabling the patient to experience positive reinforcements [85]. In addition, psychodynamic therapies or the cognitive behavioral analysis system of psychotherapy (CBASP) focussing on the experience of past and current relationships could teach patients to become connected with the depressogenic consequences of their interpersonal behavior [86, 87] potentially counteracting an upregulated SADNESS system by enabling rewarding social interactions. In addition, we found female gender to be a risk factor for early age at depression onset, which is in line with previous studies on sex differences in depression development [88].

It is also worth mentioning that we discovered slightly different methylation patterns between the variables of interest when investigating men and women separately. The expected positive association between SLEs and SADNESS was found only in men. Further, the negative association between depression severity and SEEKING was only present in women. This could mean one of four things: for one, depressive symptoms in women could differ from depressive symptoms in men in line with previous findings [89]. Second, women could have different risk factors predisposing them for depression, e.g. a more strongly expressed shutdown following separation distress as proposed by Watt and Panksepp [16]. Third, depressive symptoms could have a different impact on primary emotions as a function of sex. Fourth, power was too low to detect the association in men (we could only investigate *n* = 51 males compared to *n* = 95 females), which is supported by the low to medium size negative association between SEEKING and depression severity found in men (BDI-II: *r* = − 0.27, MADRS: *r* = − 0.30). The finding of women scoring higher on SADNESS than men is in line with previous studies reporting that women tend to naturally report higher neuroticism scores, especially in egalitarian societies [90].

Even though we did not find differences between inpatients without and with a family history of psychiatric disorders, psychiatric disorders in family members are an important factor associated with depression development and suicide [91, 92]. In addition, parents’ psychiatric disorders could be stressors affecting epigenetic mechanisms in the offspring [93]. A possible explanation for the non-significant results regarding this variable could be that we collected data on family psychiatric history by means of self-report and that patients may not correctly assess the psychopathology of family members.

Contradictory to our expectations, in the whole sample, SLC6A4 methylation was associated with neither of the investigated variables. When looking at women and men separately, we found one significantly positive association between SLC6A4 methylation factor 2 and depression severity in women. This association is in line findings of higher SLC6A4 methylation being associated with depression severity [42, 44]. There are, however, also studies reporting negative associations between SLC6A4 methylation and depression severity [42, 43, 45–47]. Depending on the specific CpG site or cluster of CpG sites examined, there may be positive or negative associations between SLC6A4 methylation, personality and depression. In line with a previous study [94] we found women to have higher SLC6A4 methylation of centric to terminal CpG sites as indexed by methylation factor 1. Since methylation of factor 1 is not associated with any of the investigated variables, this sex difference in SLC6A4 methylation does not seem to be related to SLEs or depressive symptoms. Furthermore, SLC6A4 methylation was not included in the model prediciting age at depression onset. Therefore, SLC6A4 methylation does not seem strongly related to depression, which questions the usefulness of SLC6A4 methylation as biomarker for depression onset. But again, our insights are also limited by the rather small sample of patients investigated. Beyond that, it is possible that SLC6A4 methylation was associated with depression severity at the time of onset of the first depressive episode. Furthermore, it is possible that SLC6A4 methylation differs between individuals developing a depressive episode and individuals that do not develop a depressive episode. Another interesting question is whether and when a stressor affects SLC6A4 methylation. It is possible that depression itself is stressful [95] and leaves traces in epigenetic signatures of the serotonin system of affected individuals which would make it more difficult to distinguish epigenetic mechanisms involved in depression development from signatures of depression itself. Therefore, prospective studies investigating epigenetic signatures before and after depression onset as well as during the course of the disorder are needed.

We did not find any significant association between the examined variables and 5-HTTLPR genotype (see supplementary material Table S4). Even though our sample size is too small for making conclusions on the presence or absence of a genotype effect, this is in line with the meta-analysis by Culverhouse and colleagues [32], who did not find strong evidence of a main effect or interaction of 5-HTTLPR in the development of depression. Overall, our findings do not support the notion of a strong linear association between SLC6A4 regulation and depression. However, there could still be differences in SLC6A4 methylation when comparing inpatients suffering from depression to healthy controls. Nevertheless, SLC6A4 appears to be associated with depression, but in a more complex fashion. There might be moderating factors other than sex and 5-HTTLPR that make it difficult to understand the exact function of SLC6A4 in the development and maintenance of depression. However, if the lack of a direct association between SLC6A4 or the 5-HTT and depression replicates in future studies, the mechanism of action of SSRIs and the role of the serotonin system in the development and maintenance of depression as a whole would need to be re-discussed. After all, it has been shown that neurogenesis mediates some beneficial effects of antidepressant treatment [96]. Additionally, first steps towards a joint explanation of the effectiveness of different kinds of antidepressants have already been taken and point towards a role of sphingolipid-controlled autophagy as an important target for antidepressive treatment [97]. A better understanding of the mechanism of action of antidepressants and the role of the 5-HTT in depression and antidepressive treatment could clarify the question of whether current antidepressants are effective in the treatment of depression and how they exert their antidepressive effect [98–101].

Some limitations need to be considered when interpreting the results of our study. First, methylation in whole blood samples is only a proxy for epigenetic profiles in brain tissue. Second, we cannot draw conclusions regarding the functionality of the observed alterations in SLC6A4 methylation since we did neither assess mRNA nor 5-HTT levels. Third, statistical power could be too low to detect associations between SLC6A4 methylation and age at depression onset or depression severity. A post-hoc power analysis, however, revealed that given our total sample size of *N* = 146 and *α* = .05 (two-tailed) power to detect a medium-sized effect [102] was determined to be 0.97. However, we cannot rule out the possibility that there are small associations between SLC6A4-methylation and age at depression onset. Fourth, we did not assess the timing of the stressor, which is why we cannot differentiate between early and late life stressors. Last, we assessed depression onset retrospectively. In future, studies with prospective longitudinal designs are needed to confirm and extend our results.

Taken together, we provide evidence that young age at depression onset is associated with depressive symptom severity. In addition, we found that a considerable amount of variance in depression onset can be explained by sex, the experience of SLEs and personality traits comprising high SADNESS and low SEEKING. Thus, our work can serve as starting point for future studies using a longitudinal design for the investigation of the causal role of sex, primary emotions, SLEs and epigenetic factors for depression development in young age. As the number of people suffering from depression rises, early identification of at-risk individuals is becoming increasingly important for establishing prevention interventions alleviating the burden that depression imposes on individuals, their social environment and society.

## Supplementary Information

**Additional file 1.**

## Data Availability

The datasets analyzed during the current study are not publicly available since the authors do not have permission to publish the data. However, data are available from the corresponding author on reasonable request.

## Abbreviations

5-HT: Serotonin
5-HTT: Serotonin transporter
5-HTTLPR: Serotonin transporter linked polymorphic region
AIC: Akaike information criterion
ANPS: Affective Neuroscience Personality Scales
BDI-II: Beck Depression Inventory
BMI: Body Mass Index
CLEQ: Critical Life Events Questionnaire
CpG: Cytosine-phosphate-guanine dinucleotides
DE: Dose equivalents
FDR: False discovery rate
MADRS: Montgomery Asberg Depression Rating Scale
MDD: Major Depressive Disorder
SLE: Stressful life event
SSRI: Selective serotonin reuptake inhibitor
