# Correction to: Factors related to age at depression onset: the role of SLC6A4 methylation, sex, exposure to stressful life events and personality in a sample of inpatients suffering from major depression

**DOI:** 10.1186/s12888-021-03215-0

**Published:** 2021-04-29

**Authors:** Simon Sanwald, Katharina Widenhorn-Müller, Carlos Schönfeldt-Lecuona, GenEmo Research Group, Christian Montag, Markus Kiefer

**Affiliations:** 1 Department of Psychiatry and Psychotherapy III, Ulm University, Ulm, Germany; 2 Department of Molecular Psychology, Institute of Psychology and Education, Ulm University, Ulm, Germany

**Correction to: BMC Psychiatry 21, 167 (2021)**

**https://doi.org/10.1186/s12888-021-03166-6**

Following the publication of the original article [1], the authors identified an error in the order of the authors names.

The incorrect order:

Simon Sanwald^1*^, Katharina Widenhorn-Müller^1^, Carlos Schönfeldt-Lecuona^1^, Christian Montag^2†^, Markus Kiefer^1†^ and GenEmo Research Group

The correct order:

Simon Sanwald^1*^, Katharina Widenhorn-Müller^1^, Carlos Schönfeldt-Lecuona^1^, GenEmo Research Group, Christian Montag^2†^ and Markus Kiefer^1†^

The author group has been updated above and the original article [1] has been corrected.
