# Supplementary material for: Factors related to age at depression onset: the role of SLC6A4 methylation, sex, exposure to stressful life events and personality in a sample of inpatients suffering from major depression
Source: BMC Psychiatry. 2021 Mar 25;21:167. doi: 10.1186/s12888-021-03166-6 (PMC7995700; doi:10.1186/s12888-021-03166-6)
Supplement: Supplementary file 1 — Additional file 1. [file 12888_2021_3166_MOESM1_ESM.docx]

Factors related to age at depression onset: The role of SLC6A4 methylation, sex, exposure to stressful life events and personality in a sample of inpatients suffering from major depression

^1^Simon Sanwald*, ^1^Katharina Widenhorn-Müller, ^1^Carlos Schönfeldt-Lecuona, GenEmo Research Group, ^2§^Christian Montag and ^1§^Markus Kiefer

^1^Ulm University, Department of Psychiatry and Psychotherapy III

^2^Ulm University, Department of Molecular Psychology, Institute of Psychology and Education

§ Both authors contributed equally to this work

GenEmo Research Group: Bernhard J. Connemann, Maximilian Gahr, Thomas Kammer

*Correspondence to:

Simon Sanwald

Ulm University

Department of Psychiatry

Section for Cognitive Electrophysiology

Leimgrubenweg 12, 89075 Ulm, Germany

Phone: +49 731 500 61538 Fax: +49 731 500 61542

Email: [Simon.Sanwald@uni-ulm.de](mailto:Simon.Sanwald@uni-ulm.de)

Supplementary Material

**Reliability Analysis for mean SLC6A4 methylation**

Reliability across CpG sites was good (*α* = .82). Table S1 shows item-rest correlations.

**Table S1.**

Item-rest correlations and Cronbach’s α (if item dropped) for the CpG units in the examined region of the SLC6A4 gene.

|  |  | If item dropped |
| --- | --- | --- |
| CpG unit | item-rest correlation | Cronbach's α |
| CpG 1 | 0.299 | 0.816 |
| CpG 3 | 0.207 | 0.817 |
| CpG 4 | 0.348 | 0.814 |
| CpG 5.6 | 0.134 | 0.822 |
| CpG 8.9 | 0.278 | 0.816 |
| CpG 10 | 0.191 | 0.817 |
| CpG 11-15 | 0.202 | 0.817 |
| CpG 16.17 | 0.228 | 0.817 |
| CpG 18.19 | 0.245 | 0.816 |
| CpG 27 | 0.251 | 0.817 |
| CpG 28 | 0.296 | 0.816 |
| CpG 29.30 | 0.255 | 0.817 |
| CpG 31.32 | 0.268 | 0.817 |
| CpG 39-42 | 0.367 | 0.812 |
| CpG 43.44 | 0.267 | 0.815 |
| CpG 45 | 0.539 | 0.809 |
| CpG 46 | 0.086 | 0.820 |
| CpG 47.48 | 0.636 | 0.804 |
| CpG 52 | 0.273 | 0.815 |
| CpG 53.54 | 0.377 | 0.813 |
| CpG 57.58 | 0.582 | 0.801 |
| CpG 60 | 0.358 | 0.812 |
| CpG 61-63 | 0.316 | 0.814 |
| CpG 64 | 0.567 | 0.801 |
| CpG 65 | 0.472 | 0.817 |
| CpG 66 | 0.578 | 0.800 |
| CpG 68 | 0.516 | 0.804 |
| CpG 69 | 0.540 | 0.803 |
| CpG 70.71 | 0.445 | 0.809 |

**Principal Component Analysis**

At first, we excluded CpG units 10 and 11-15 since these units had very low correlations with all other CpG units.

**Table S2.**

Factor loadings of single CpG units for the varimax-rotated factor solution.

|  | Factor 1 | Factor 2 |
| --- | --- | --- |
| CpG_1 |  | 0.643 |
| CpG 3 |  |  |
| CpG 4 |  | 0.675 |
| CpG 5.6 |  |  |
| CpG 8.9 |  | 0.493 |
| CpG 16.17 | | 0.715 |
| CpG 18.19 | | 0.556 |
| CpG 27 |  |  |
| CpG 28 |  | 0.520 |
| CpG 29.30 | | 0.532 |
| CpG 31.32 | 0.432 |  |
| CpG 39-42 | 0.388 |  |
| CpG 43.44 | |  |
| CpG 45 | 0.670 |  |
| CpG 46 |  |  |
| CpG 47.48 | 0.667 |  |
| CpG 52 |  | 0.521 |
| CpG 53.54 | | 0.376 |
| CpG 57.58 | 0.674 |  |
| CpG 60 | 0.489 |  |
| CpG 61-63 | 0.514 |  |
| CpG 64 | 0.748 |  |
| CpG 65 | 0.431 |  |
| CpG 66 | 0.416 | 0.378 |
| CpG 68 | 0.607 |  |
| CpG 69 | 0.709 |  |
| CpG 70.71 | 0.751 |  |

**Correlations**

After FDR correction, there was only one significant and negative association between CpG 31.32 and SEEKING.

**Table S3.**

Spearman’s correlation coefficients for the associations of CpG units with the variables of interest in the whole group.

|  | BDI-II | MADRS | CLEQ | SEEKING | SADNESS | age at onset |
| --- | --- | --- | --- | --- | --- | --- |
| CpG_1 | -0.12 | 0.01 | -0.06 | 0.09 | -0.03 | -0.01 |
| CpG_3 | -0.04 | 0.01 | -0.06 | -0.07 | -0.01 | 0.06 |
| CpG_4 | -0.05 | 0.08 | 0.04 | 0.07 | 0.07 | -0.06 |
| CpG_5.6 | -0.07 | 0.02 | -0.08 | -0.06 | -0.03 | 0.02 |
| CpG_8.9 | 0.04 | 0.17 | 0.00 | -0.02 | 0.06 | 0.04 |
| CpG_10 | -0.12 | -0.04 | -0.09 | 0.05 | 0.05 | 0.04 |
| CpG_11-15 | -0.01 | 0.04 | -0.04 | -0.02 | 0.02 | 0.10 |
| CpG_16.17 | -0.07 | 0.03 | -0.10 | 0.05 | -0.01 | 0.18 |
| CpG_18.19 | 0.07 | 0.13 | 0.01 | 0.05 | 0.07 | 0.03 |
| CpG_27 | -0.11 | -0.01 | -0.13 | -0.03 | -0.13 | 0.06 |
| CpG_28 | 0.12 | 0.16 | 0.06 | 0.02 | 0.11 | -0.04 |
| CpG_29.30 | -0.04 | 0.08 | -0.15 | 0.04 | -0.10 | 0.02 |
| CpG_31.32 | 0.01 | 0.02 | 0.02 | -0.26* | -0.16 | -0.01 |
| CpG_39-42 | -0.04 | 0.02 | 0.01 | 0.02 | -0.09 | 0.02 |
| CpG_43.44 | -0.07 | -0.08 | -0.18 | -0.05 | -0.10 | 0.21 |
| CpG_45 | -0.09 | -0.04 | 0.10 | -0.00 | -0.04 | -0.02 |
| CpG_46 | -0.01 | 0.13 | 0.11 | 0.07 | -0.12 | -0.02 |
| CpG_47.48 | 0.14 | 0.18 | 0.02 | 0.00 | 0.07 | -0.03 |
| CpG_52 | -0.06 | 0.09 | -0.11 | 0.06 | 0.05 | -0.04 |
| CpG_53.54 | -0.04 | 0.01 | -0.15 | -0.09 | 0.03 | 0.05 |
| CpG_57.58 | 0.01 | 0.07 | 0.00 | -0.01 | 0.08 | -0.00 |
| CpG_60 | 0.19 | 0.20 | -0.09 | -0.15 | -0.03 | -0.07 |
| CpG_61-63 | 0.05 | 0.01 | -0.07 | -0.00 | -0.01 | 0.01 |
| CpG_64 | 0.12 | 0.08 | -0.02 | -0.11 | 0.00 | 0.02 |
| CpG_65 | 0.04 | 0.10 | -0.14 | -0.01 | 0.07 | -0.03 |
| CpG_66 | 0.03 | 0.08 | -0.01 | -0.05 | 0.07 | -0.01 |
| CpG_68 | 0.12 | 0.07 | 0.07 | 0.11 | 0.05 | -0.22 |
| CpG_69 | 0.02 | 0.02 | 0.08 | -0.06 | 0.05 | -0.17 |
| CpG_70.71 | 0.09 | 0.07 | -0.04 | -0.08 | 0.00 | -0.13 |

*Note.* Covariates: age and BMI. * *p_BH_* < .05.

**Effects of 5-HTTLPR rs25531 genotype**

We did not find any significant effect of genotype for any of the investigated variables even before controlling FDR (Table S7). This was true for parametric as well as non-parametric test statistics.

**Table S4.**

Group differences between genotype groups.

|  | Test | Statistic | *df* | *p* |
| --- | --- | --- | --- | --- |
| Age | Welch | 0.451 | 75.833 | 0.653 |
|  | Mann-Whitney | 2238 |  | 0.675 |
| BMI | Welch | 0.899 | 63.992 | 0.372 |
|  | Mann-Whitney | 2262 |  | 0.599 |
| Alcohol (grams/day) | Welch | 0.426 | 47.374 | 0.672 |
|  | Mann-Whitney | 1703.5 |  | 0.238 |
| Cigarettes/day | Welch | -0.108 | 90.179 | 0.915 |
|  | Mann-Whitney | 2101.5 |  | 0.879 |
| Timespan | Welch | 0.445 | 73.430 | 0.658 |
|  | Mann-Whitney | 2231 |  | 0.697 |
| DE antidepressants | Welch | 1.208 | 66.866 | 0.231 |
|  | Mann-Whitney | 1827 |  | 0.075 |
| DE neuroleptics | Welch | 0.373 | 65.201 | 0.710 |
|  | Mann-Whitney | 1974 |  | 0.678 |
| BDI-II | Welch | 0.191 | 73.468 | 0.849 |
|  | Mann-Whitney | 1854.5 |  | 0.900 |
| MADRS | Welch | -0.022 | 75.771 | 0.983 |
|  | Mann-Whitney | 1936.0 |  | 0.900 |
| CLEQ | Welch | 0.595 | 57.172 | 0.554 |
|  | Mann-Whitney | 1936 |  | 0.721 |
| SEEKING | Welch | 1.216 | 94.147 | 0.227 |
|  | Mann-Whitney | 2042.5 |  | 0.330 |
| SADNESS | Welch | -0.310 | 84.749 | 0.757 |
|  | Mann-Whitney | 1728.0 |  | 0.517 |

*Note.* DE = dose equivalents.
